# 

**Published:** 1883-05

**Authors:** 


### American Medical Association.

The thirty-fourth annual session will be held in Cleveland, Ohio, on Tuesday, Wednesday, Thursday and Friday, June 5, 6, 7, 8, 1883, commencing on Tuesday, at 11 o'clock A.M.

"The delegates shall receive their appointment from permanently organized State Medical Societies, and such County and District Medical Societies as are recognized by *representation in their respective State Societies*, and from the Medical Department of the Army and Navy, and the Marine Hospital Service of the United States.

"Each State, County, and District Medical Society entitled to representation shall have the privilege of sending to the Association one delegate for every ten of its regular resident members, and one for every additional fraction of more than half that number: *Provided*, however, that the number of deligates for any particular State, territory, county, city, or town shall not exceed the ratio of one in ten of the resident physicians who may have signed the Code of Ethics of the Association."

SECTIONS.

"The Chairman of the several Sections shall prepare and read, in the general sessions of the Association, papers on the advances and discoveries of the past year in the branches of science included in their respective sections. * * * * *"

*By-Laws*, Art. II., Sect. 4.

Practice of Medicine, Materia Medica, and Physiology: Dr. J. H. Hollister, Chicago, Ill., Chairman. Dr. J. G. Lee, Philadelphia, Secretary.

Obstetrics and Diseases of Women and Children: Dr. J. K. Bartlett, Milwaukee, Wis., Chairman; Dr. G. A. Moses, St. Louis, Mo., Secretary.

Surgery and Anatomy: Dr. W. F. Peck, Davenport, Iowa, Chairman; Dr. P. F. Eve, Nashville, Tenn., Secretary.

State Medicine: Dr. Foster Pratt, Kalamazoo, Mich., Chairman; Dr. T. L. Neal, Dayton, Ohio, Secretary.

Ophthalmology, Otology, and Laryngology: Dr. A. W. Calhoun, Atlanta, Ga, Chairman; Dr. Carl Seiler, Philadelphia, Secretary.

Diseases of Children: Dr. R. F. Blount, Wabash, Ind., Chairman; Dr. J. H. Sears, Waco, Texas, Secretary.

Oral and Dental Surgery: Dr. D. H. Goodwillie, New York City, Chairman; Dr. T. W. Brophy, Chicago, Ill., Secretary.

A member desiring to read a paper before any Section should forward the paper, or its *title* and *length* (not to exceed twenty minutes in reading), to the Chairman of the Committee of Arrangements at least one month before the meeting.—*By-Laws.*

*Committee of Arrangements.*—Dr. X. C. Scott, 393 Euclid Avenue, Cleveland, Ohio, Chairman.

### Examinations.

The Illinois State Board of Dental Examiners will meet at the new Demming House, in Decatur, at 9 o'clock A. M., Monday, May 7, 1883. Candidates for examination must present themselves at that time. GEO. H. CUSHING, Secretary.

### Illinois State Dental Meeting.

The annual meeting of this body will be held on Tuesday, May 8th, at Decatur, begining at 10 o'clock A.M.

The meetings of this society are always good, interesting, and profitable, at least for all who are willing to learn.

There is usually a large number in attendance, and a larger attendance will probably be present this year. The profession of Illinois has very interesting matters in hand at present. A general invitation is always extended to the profession, not only in the State, but outside as well. There is room, and large hearts for all.

### CONTENTS.

COMMUNICATIONS.

THE DENTAL REGISTER,

A MONTHLY MAGAZINE DEVOTED TO THE INTERESTS OF THE

**DENTAL PROFESSION.**

*Terms Reduced to* $2.00 *Per Year. Single Copies,* 25 *Cents.*

**EDITED BY PROF. J. TAFT, D.D.S.**

AND

***Published by SPENCER & CROCKER, Ohio Dental Depot.***

The volume commences in January and closes in December.

The REGISTER being issued monthly is always fresh, therefore Indispensable to the practitioner who desires to keep posted up with the new inventions and improvements which are daily developed. Neither expense nor effort will be spared to give value and variety to its contents. Articles will be illustrated with well executed engravings whenever they will add to the interest or assist to explanation.

Its extensive circulation and frequency of issue make it one of the BEST DENTAL ADVERTISING MEDIUMS in the United States.

All subscriptions must begin with January or July.

Local or special notices, five lines or less, will be inserted for $3 each insertion; over five lines, 50 cts. per line.

All advertising bills payable in advance, or at furthest, after the issue of the first number containing the advertisement. All transient advertisements to be paid for in advance.

☞CONTRIBUTIONS SOLICITED.

Exclusively Editorial Communications should be addressed to the Editor.

The latest number of the REGISTER may be ordered with or without other goods at any time, and all letters should be addressed to

**SPENCER & CROCKER, Ohio Dental Depot, Cincinnati, O.**

